# Renal Metastasis of Adenocarcinoma of the Colon

**DOI:** 10.1155/crip/5320139

**Published:** 2025-09-16

**Authors:** Ramin Saadaat, Saifullah, Mohammad Asef Adelyar, Jamshid Abdul-Ghafar, Mohibullah Rahmani, Esmatullah Esmat, Ahmed Maseh Haidary, Haider Ali Malakzai

**Affiliations:** ^1^Department of Pathology and Clinical Laboratory, French Medical Institute for Mothers and Children (FMIC), Kabul, Afghanistan; ^2^Department of Internal Medicine, French Medical Institute for Mothers and Children (FMIC), Kabul, Afghanistan; ^3^Department of General Surgery, Kabul University of Medical Science (KUMS), Kabul, Afghanistan

**Keywords:** case report, colon cancer, colorectal, kidney metastasis, surgical treatment

## Abstract

**Introduction:** Metastasis is a critical factor in colorectal cancer (CRC) outcomes, with 22% of patients presenting with metastasis at diagnosis and an eventual 70% experiencing it. This report highlights a rare case of ascending colon adenocarcinoma with metastasis to the kidney, underscoring the diverse and complex nature of CRC progression.

**Case Presentation:** A 60-year-old man presented with abdominal discomfort, constipation, and rectal bleeding after colonoscopy revealed a colon mass, leading to a diagnosis of adenocarcinoma after colonoscopic biopsy. Initially without distant metastasis, he underwent four cycles of chemotherapy, but follow-up imaging 6 months later showed liver and renal metastases, prompting a colectomy and nephrectomy. Pathological examination confirmed moderately differentiated adenocarcinoma in both the colon and kidney, with staging indicating advanced disease, and the patient succumbed to his illness shortly after surgery.

**Clinical Discussion:** Metastatic carcinomas to the kidney are uncommon, with CRC metastasis being particularly rare, as evidenced by a limited number of cases in the literature. Typically originating from primary tumors in the lung, liver, and gastrointestinal tract, renal metastases often present as well-defined lesions, complicating the differentiation from primary renal cancers. Our case highlights a solitary, well-circumscribed renal metastasis from CRC, emphasizing the diagnostic challenges and the need for careful evaluation in patients with known malignancies.

**Conclusion:** Metastatic carcinoma of the colorectal tract is very rarely reported to the kidney, it does so at a higher stage of the disease with systemic disease and has a poor outcome for the patient.

## 1. Introduction

Globally, cancer is a major health challenge and is responsible for almost one in six deaths (16.8%) and one in four deaths (22.8%) from noncommunicable diseases (NCDs). According to GLOBOCAN cancer data, among cancers from all sites of the body, more than 1.9 million (9.6% of all body site cancer) colorectal cancer (CRC) new cases and more than 90 thousand (9.3% of all body site cancers) deaths were estimated to occur in 2022 globally. This data demonstrates that around one in 10 cases of cancers belongs to CRC and will rank in third position based on cancer incidence; however, in terms of mortality, CRC will rank in second position [1]. The mortality of CRC is mainly related to its metastasis, and at the time of initial diagnosis, up to 22% will have metastasis, and eventually, around 70% will experience the metastasis [2]. The overall prognosis for patients with metastatic CRC is poor; the relative 5-year survival rate is 14%, while the rates for patients with regional and localized CRC in the United States are 71% and 90%, respectively [3]. The first and very common site for CRC metastasis is the liver, and in 60% of cases, it will happen, and the lung is the second most common site for metastasis [4]. CRC metastasis to the kidney is very rare [5], and here, we report a case of ascending colon adenocarcinoma with metastasis to the kidney.

## 2. Case Presentation

**Figure 1:**
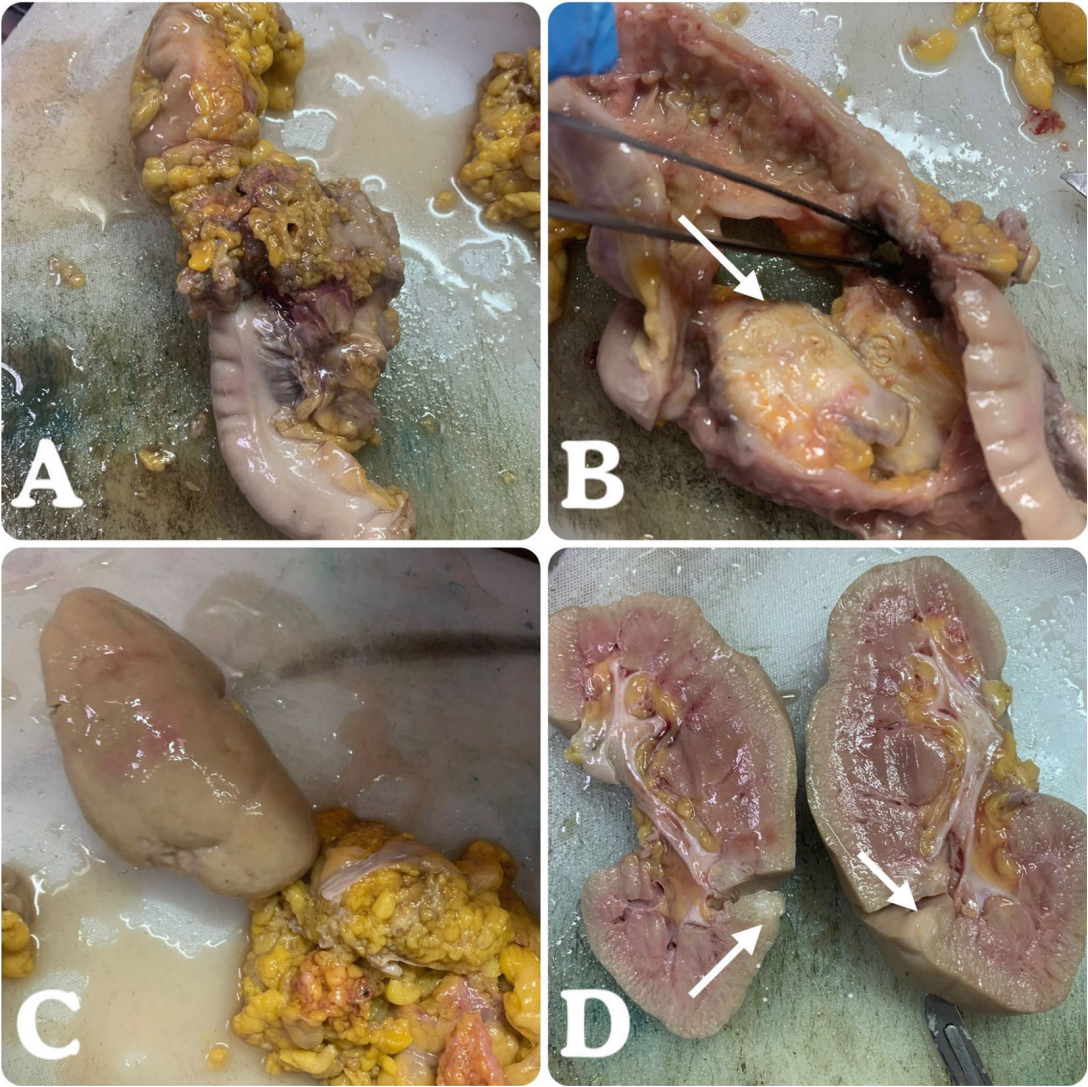
Gross images of the intestinal segment and kidney. The large intestine segment with attached pericolic fat tissue is shown (A). The lumen of the intestine reveals a large circumferential mass (arrow) (B). The nephrectomy specimen with a smooth surface is shown (C). After bivalving the kidney, the lower pole displays a round mass in the cortical portion (arrows) (D).

**Figure 2:**
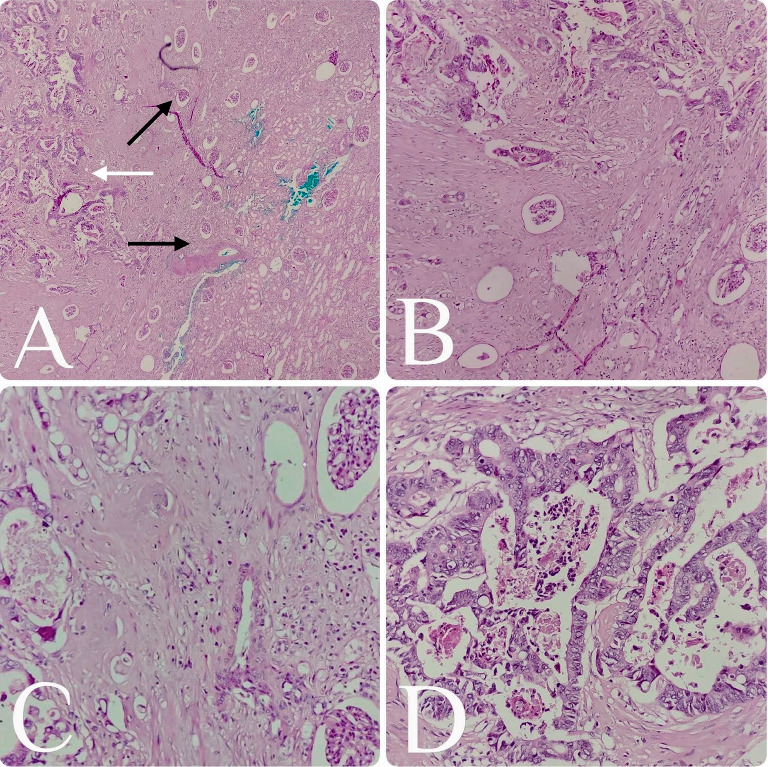
H&E staining of the tissue. At 4x magnification, the renal parenchyma is shown on the right side (black arrows), and metastatic adenocarcinoma is on the left side (white arrow) (A). At 10x magnification, the renal parenchyma is located in the lower right and mid portions of the image, while metastatic adenocarcinoma is present on the left and upper sides (B). At 20x magnification, the renal parenchyma, composed of normal glomeruli, is seen on the right side of the image, and metastatic adenocarcinoma is on the left (C). At 40x magnification, the tumor consists of complex glandular proliferation with highly atypical and pleomorphic lining cells exhibiting prominent nucleoli (D).

A 60-year-old was presented to the hospital with a complaint of abdominal discomfort, constipation for a few months, and recently with rectal bleeding. The man was a government office worker, educated, and living in a province and had an average socioeconomic status with oral snuff use for many years. This patient had no other health issues or complaints and no cancer history in his family, according to his explanation. Colonoscopy was performed for him, and a mass in the descending colon was detected, and after biopsy examination, it was diagnosed as adenocarcinoma. At the time of diagnosis, no distant metastasis was noted based on radiological evaluations. The oncology team started a few cycles of chemotherapy for the patient, but after 6 months, when it was evaluated with follow-up radiology, the patient had liver and renal metastasis. Therefore, surgery was performed for the patient, consisting of colectomy and nephrectomy. We received a segment of colon measuring 25 cm in length with a few attached pericolic fat tissues and no complete mesocolic excision and a kidney measuring 13 × 8 × 5 cm Figure 1A,C. The colon showed a bulging mass and perforation. On opening the colon through the antimesenteric border, there was a circumferential, ulcerative gray–white mass measuring 8 × 6 × 5 cm. On cut sections, it revealed a gray–white fibrotic and infiltrative appearance with perforation of the wall of the intestine and extending to the pericolic fat tissue. The pericolic fat tissue also showed a small tumor deposition measuring 1 × 0.5 × 0.5 cm (Figure 1B). The pericolic fat dissection was done, and only four lymph nodes were found. The microscopic examination of the colon mass showed moderately differentiated adenocarcinoma reaching the serosa surface. The kidney on gross examination revealed a round nodular mass in the cortex of the lower pole, measuring 1 × 1 × 0.8 cm (Figure 1D). The microscopic examination showed a moderately differentiated adenocarcinoma composed of cribriform and complex glandular proliferations, and the neoplastic cells were pleomorphic with enlarged hyperchromatic nuclei and prominent nucleoli. The sections of kidney tissue also showed the same morphology of neoplasm, and it is consistent with metastatic adenocarcinoma from the colon (Figures 2A, 2B, 2C, and 2D). The pathological staging of the patient, with deposition in pericolic fat tissue, indicated that all four submitted lymph nodes were free of tumor, and distant metastasis to the kidney was assigned as IVA (pT4aN1cM1a). After the surgery, the patient received some supportive therapy and finally expired after a month.

## 3. Discussion

Metastatic carcinomas to the kidney are not very common and have not been studied much in the literature. Mostly, metastatic carcinoma to the kidney originates in the lung, liver, and gastrointestinal tract. In the literature, renal metastasis from CRCs is extremely rarely estimated (6). In 1979, a study was performed on 11,378 autopsies, and the result showed 816 (7.2%) renal metastases. The most common primary tumors among the metastases to the kidney, in a decreasing sequence of frequency, were lung, breast, skin (melanoma), and tumors of the genitourinary, gastrointestinal, and gynecologic tracts, respectively [6]. However, in autopsy studies, metastatic tumors to the kidney are reported as lower up to 3% and higher up to 30%, respectively [7, 8].

Literature reviewed by Dulskas et al. in 2015 identified 15 cases of CRC metastasis to the kidney, including their own case [5]. Although metastatic carcinomas to the kidney have similar features to those in primary carcinomas in radiology and gross examinations, multiple lesions and involvement of the bilateral kidney can be more in favor of metastatic rather than primary carcinomas [8]. In our case, the metastatic lesion was small, unilateral, and solitary. The time interval between the diagnosis of the primary tumor and metastasis is various. In one cohort study on metastatic carcinomas to the kidney, a reported median time of 3.1 years from primary tumor diagnosis to metastatic lesion was noted; however, the range was broad from months to 26 years, and in 19% of the total cases in this study, the time interval between the primary tumor and metastatic tumor was more than 10 years [9]. The radiological characteristics of renal metastases are indicative of their pathological involvement. Typically, these lesions present as well-defined rounded masses, though infiltrative lesions can sometimes be observed. In the case that we are presenting, the metastatic lesion was identified as a small, well-circumscribed mass located in the renal cortex [10].

The colon is drained by the portal venous system, while metastasis to the brain, spleen, and kidneys occurs through arterial dissemination. This mode of dissemination is uncommon in colon cancer, with only 3% of all colon cancer metastases resulting from arterial pathways [11].

Patients with a known extrarenal primary malignancy who present with a solitary renal lesion pose a significant diagnostic challenge, as the potential for synchronous primary renal cancer must be taken into account. In a retrospective study involving 763 patients who underwent surgery for renal cell carcinoma, 209 of these patients (27.4%) were found to have second primary malignancies. The five most common second malignancies identified were prostate, breast, colon, and bladder cancers, along with non-Hodgkin's lymphoma [12, 13]. Mostly, a specific treatment is not required for renal metastasis as it generally occurs in a more advanced stage of the disease; however, synchronous involvement of the kidney during the initial diagnosis may be followed by nephrectomy. In our case, the nephrectomy was done for the patient, maybe for the confirmation of the metastatic lesion.

## 4. Conclusion

Metastatic carcinoma to the kidney is rare, particularly from CRCs, with only a few documented cases in the literature. While renal metastases often originate from primary tumors in the lung, liver, and gastrointestinal tract, the characteristics of these lesions can mimic those of primary renal cancers, complicating diagnosis. In our case, the solitary, well-circumscribed renal metastasis underscores the need for careful evaluation in patients with known malignancies presenting with renal lesions.

## Data Availability

All the available data have been included in the manuscript.

## Patient Perspective

The patient's attendant stated that it would be useful to publish and share this case with other healthcare workers, to enable better understanding and correct diagnosis of such rare cases that would cause a specific treatment and follow-up.
